# Control of parasitic diseases in aquaculture

**DOI:** 10.1017/S0031182022001093

**Published:** 2022-12

**Authors:** Kurt Buchmann

**Affiliations:** Laboratory of Aquatic Pathobiology, Department of Veterinary and Animal Sciences, Faculty of Health and Medical Sciences, University of Copenhagen, Frederiksberg C., Denmark

**Keywords:** Aquaculture, control, disease, fish, parasite

## Abstract

Finfish aquaculture in freshwater and marine environments is continuously expanding globally, and the potential for a substantial further increase is well documented. The industry is supplying fish products for human consumption to the same extent as capture fisheries, and new fish species for domestication are still being selected by the industry. The challenge faced by all aquacultured species, classical and novel, is the range of pathogens associated with each new fish type. A fish host in its natural environment carries a series of more or less specific parasites (specialists and generalists). Some of these show a marked ability to propagate in aquaculture settings. They may then elicit disease when infection intensities in the confined aquaculture environment reach high levels. In addition, the risk of transmission of parasites from aquaculture enterprises to wild fish stocks adds to the parasitic challenge. Control programmes of various kinds are needed and these may include chemotherapeutants and medicines as the farmer's first and convenient choice, but mechanical, biological, immunological and genetic control methods are available solutions. New methods are still to be developed by scrutinizing the life cycle of each particular parasite species and pin-pointing the vulnerable stage to be targeted. As parasites exhibit a huge potential for adaptation to environmental changes, one must realize that only one approach rarely is sufficient. The present work therefore elaborates on and advocates for implementation of integrated control strategies for diseases caused by protozoan and metazoan parasites.

## Graphical abstract

**Figure:**
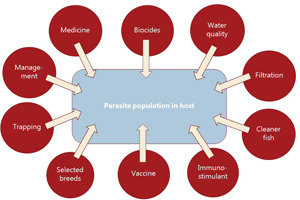

## Introduction

Finfish aquaculture in freshwater and marine environments is continuously expanding globally (FAO, 2020) and the potential for a substantial further increase is well documented in the marine environment (Gentry *et al*., 2017). Freshwater aquaculture of fish dominates at present in Asia and particularly in China and Indonesia, but expansion of this branch in Europe and the Americas is possible through application of recirculation technology, which receives attention and increased investment capital. The aquaculture industry is supplying fish products for human consumption to the same extent as capture fisheries, and new fish species for domestication are still being selected by the industry (FAO, 2020). The challenge faced by all aquacultured species, classical and novel, is the range of pathogens associated with each new fish type. A fish host in its natural environment carries a series of more or less specific parasites (specialists and generalists) (Woo *et al*., 2020). Even if disease-free fish are used for stocking, disease problems may arise in a fish production system, provided the farm becomes exposed to pathogens (water intake from external sites). Parasite stages from wild fish populations can infect and propagate on the aquacultured fish (spill-over from the environment). As some of these organisms show a marked ability to propagate in aquaculture settings, they may elicit disease as infection intensities increase in the confined aquaculture environment. This will challenge the health and welfare of the fish and the economy of the aquaculture enterprises (Shinn *et al*., 2015). The risk of transmission of parasites from the aquaculture enterprises to wild fish stocks (back-spill) adds to the need for initiation of control programmes in order to protect the original endemic fish populations. Chemotherapeutants and medicines may be the farmer's first and convenient choice but mechanical, biological, immuno-prophylactic and genetic control methods are available as sustainable solutions. The present study outlines control possibilities for various parasitic groups of importance including oomycetes (*Saprolegnia*), protozoans (amoebae, flagellates, ciliates) and metazoans (myxozoans, monogeneans, digeneans, cestodes, nematodes, crustaceans) and advocates for an integrated control strategy due to the remarkable adaptivity of parasites. As background for the assessments and recommendations, a selection of relevant published scientific articles were included. Those studies are elaborating on various methods used to control parasitic infections in aquacultured fish with focus on chemotherapeutants, biocides, herbal extracts, medicines, mechanical methods and immunoprophylactic methods including vaccination. Combination of the methodologies may be considered if an integrated control strategy is to be implemented. The different legislation on usage of biocides and medicines in different countries may complicate their application as many of the compounds may be licensed in some countries but not in others.

## Chemotherapeutants and biocides

A series of chemicals with a problematic toxicity profile are well known in aquaculture suffering from ectoparasitic infections. The organic dye malachite green was previously applied in even low concentrations for elimination of oomycetes (e.g. *Saprolegnia*) from fish eggs and fish larvae. It also effectively kills parasitic ciliates such as *Ichthyophthirius multifiliis* and flagellates such as *Ichthyobodo*, *Piscinoodinium* and *Amyloodinium*. However, concerns on the toxicity of malachite green were raised early (Alderman, 1985). Studies have shown that the compound and its metabolite leucomalachite green are carcinogenic and genotoxic (EFSA, 2016). Following the ban of malachite green several decades ago, other chemicals with some, but lower, efficacy were used in increasing amounts (Rintamäki *et al*., 2005). A range of insecticides (malathion and parathion) were previously used to eradicate crustacean parasites (Kabata, 1985), but the environmental issues, including toxicity to fish and workers, limit their application.

### Sodium chloride and freshwater

Ectoparasites on freshwater fish may be eliminated by immersion of the infected host into high NaCl concentrations. Similarly, marine parasites succumb when exposed to freshwater dependent on their ability to adjust to the change of salinity. Parasites as free-living invertebrates may show different tolerance to changing salinities. Thus, some are euryhaline and others stenohaline. The osmotic stress induced by a change of salinity may kill a range of protozoans (amoebae, flagellates, ciliates) and metazoans (monogeneans). Freshwater treatments are regularly applied to reduce populations of marine amoebae such as *Neoparamoeba perurans* on gills causing amoebic gill disease (AGD) in maricultured Atlantic salmon (Nowak, 2012). The treatment of white spot disease caused by trophonts of the freshwater ciliate *Ichthyopthirius multifiliis* is more complicated. The parasite is in principle not an ectoparasite, due to its location in the epidermis, where it is covered by a hyperplasic epithelium. It is therefore protected against osmotic stress in the host tissue. In order to eliminate this parasite in a fish farm system, the free-living stages (tomonts, tomocysts, theronts) must be targeted. This may be achieved by sustaining a high (10 ppt) concentration over 10 days at temperatures over 20°C, whereby all trophonts in the fish surface will have sufficient time to escape into the fish tank water. The high salinity will prevent development of the tomont, *via* the tomocyst stage, into infective theronts, whereby the life cycle is broken and the parasite population exhausted (Li and Buchmann, 2001). The corresponding marine species *Cryptocaryon irritans* may be controlled by a similar strategy but by use of seawater diluted (up to 1:3) with distilled water (Cheung *et al*., 1979).

### Formalin and chloramine T

Administration of formalin directly to fish tank water containing live infected fish is currently used in conventional farms and even in some recirculated systems (Madsen *et al*., 2000; Noga, 2012). Such bath treatments with the chemical in concentrations around 20–50 mg L^−1^ remove epibionts (sessile ciliates, flagellates, amoebae) (Buchmann and Bresciani, 1997; Noga, 2012) including *Amyloodinium* (Noga, 2012), *Ichthyobodo* (Jaafar *et al*., 2013) from fish surfaces, monogeneans from fish skin (Buchmann and Kristensson, 2003), gills (Buchmann, 1988) and kill infective free-living stages of e.g. *Ichthyophthirius* and *Diplostomum* (Larsen *et al*., 2005) in the fish tank water. In addition, it reduces the bacterial concentration and the infective stages of various pathogens. The bath treatment initiates a stress response in the fish, which can be measured as a surge of plasma cortisol (Jørgensen and Buchmann, 2007) and a general upregulation of proinflammatory cytokines in skin and gills (Mathiessen *et al*., 2021*b*). As the compound is allergenic and carcinogenic it is considered a human health hazard. Chloramine T has been widely used as bath treatment against similar ectoparasites but due to lack of approval by authorities the application is restrained (Lasee, 1995).

### Copper sulphate and potassium permanganate, iron and organic acids

Another widely used compound is copper sulphate with corresponding lethal effects on ectoparasites and or external infective stages (Lasee, 1995; Noga, 2012). It has documented toxic effects on *Ichthyophthirius*, *Ichthyobodo*, *Amyloodinium* and the crustacean parasite *Argulus*. Potassium permanganate has been used for similar purposes (Lasee, 1995; Straus and Griffin, 2001; Noga, 2012). Environmental concerns due to its effect on free-living organisms, including algae, may constrain approval, licensing and thereby usage in aquaculture facilities.

### Hydrogen peroxide (H_2_O_2_) and H_2_O_2_-releasing compounds

Hydrogen peroxide, sodium percarbonate and peracetic acid are potent oxidizing agents, which are widely applied in aquaculture as replacement for malachite green and formalin (Rach *et al*., 2000; Meinelt *et al*., 2009; Straus and Meinelt, 2009; Bruzio and Buchmann, 2010; Jaafar *et al*., 2013). The compounds are used for bathing of infected fish and interact with and effectively kill various ectoparasites and external stages such as theronts of *Ichthyophthirius* and *Ichthyobodo necator*. It is also applied in Mediterranean mariculture enterprises, including seabream aquacultures, suffering from gill infections caused by the monogenean *Sparicotyle chrysophrii* (Sitjà-Bobadilla *et al*., 2006). Further, hydrogen peroxide has been used for treatment of Japanese tiger puffer (*Tagifugu rubriceps*) suffering from infections of the branchial cavity wall with the diclidophorid gill monogenean *Heterobothrium okamotoi* (Ogawa and Yokoyama, 1998). The compound has also been widely applied to remove salmon lice from the surface of Atlantic salmon, but efficacy has decreased over time due to selection of partly H_2_O_2_**-**resistant parasite strains (Helgesen *et al*., 2015).

### Plant extracts

Extensive work has been conducted on the usage of plant extracts to control fish diseases, including those caused by bacterial infections (Zheng *et al*., 2009; Diler *et al*., 2017) and ectoparasites (Madsen *et al*., 2000; Tedesco *et al*., 2020). The volatile molecules in *Allium*, *Thymus*, *Origanum* and *Coriander* were found to have a short-term effect both *in vitro* and *in vivo* when screened for effects against *Ichthyophthirius* in rainbow trout (Mathiessen *et al*., 2021*a*), and correspondingly *Origanum* extracts were found lethal to *Trichodina* and *Ichthyobodo* (Mizuno *et al*., 2018). In feed application of Chinese herbal medicines such as ginger (*Zingiber officinale*) also showed a significant reducing effect on *I. multifiliis* infection in grasscarp (Lin *et al*., 2016). The number of potential parasiticides in plants is high and a range of studies have documented effects of 18 compounds against the parasitic dinoflagellate *Amyloodinium ocellatum* (Tedesco *et al*., 2020). Several others may have a potential for future licensing, but unfortunately a large part of the tested substances exhibited toxic effects in cell cultures. Functional feeds (containing plant extracts, organic acids and yeast constituents) for gilthead seabream were shown to partly counteract the pathology induced by *Enteromyxum leei* (Palenzuela *et al*., 2020). Besides the direct toxic effect of the molecules on the parasites and a possible immunostimulatory effect on the host (Lin *et al*., 2016; Mathiessen *et al*., 2021*b*), it is worthwhile to consider alternative mechanisms exerted by the herbal extracts, as they may disrupt the host-seeking behaviour of certain parasites and thereby prevent infections (O'Shea *et al*., 2016).

### Bacterial surfactants

Unicellular parasites, such as ciliates, are sensitive to a surfactant released by the bacterium *Pseudomonas* H6. *In vitro* exposure of *Ichthyophthirius* theronts, tomonts and tomocysts demonstrated a full lethal effect of the compound, even when used in low concentrations (Al-Jubury *et al*., 2018). Follow-up *in vivo* work showed that the compound in a concentration of 10 mg L^−1^ in a fish tank with a high concentration of infective theronts effectively prevented infection of rainbow trout (Li *et al*., 2022). The low toxic effect of the surfactant on trout (Mathiessen *et al*., 2021*b*) and to other ecosystem organisms (cyanobacteria, green algae, crustaceans and zebrafish) (Korbut *et al*., 2022) suggests that the product should be further assessed for a possible future application as a parasiticide in aquaculture.

### Medicines

The classical approach to parasite control is to apply various medicines as antiparasitic agents (Picon-Camacho *et al*., 2012), but a set of rules and legislation must be observed when parasitic infections in fish are to be treated with medicines. This applies for both preliminary investigational and validation studies before licensing and when administrating the licensed products (Sommerville *et al*., 2016). Before initiating treatments at farm level, a specific diagnosis should be stated and a prescription made by a veterinarian. The drug should be licensed in the particular country in which the treatment is planned. Several medicines with a known antiparasitic effect have been banned in animal production of various reasons.

### Nitroimidazoles

The group of drugs banned include, e.g. for the group of nitro-imidazoles, such as metronidazole, secnidazole and dimetridazole, although they are highly effective against flagellates (*Spironucleus vortens*) (Sangmaneedet and Smith, 1999) and ciliates (*Ichthyophthirius*) (Tokşen and Nemli, 2010). Usage of these drugs for production animals was banned in the European community for decades due to lack of needed documentation (safety, residual levels). Drugs which are licensed for one host species may in several countries be applied also to treat corresponding infections in other hosts, provided that sufficient documentation for efficacy against the disease and low toxicity to the host are available. In that case the prescription is given according to cascade rules.

### Anticoccidials

Toltrazuril (brand name Baycox^®^) is an anticoccidial which was mentioned as a parasiticide by Schmahl *et al*. (1989) following experimental *in vitro* studies with various ciliate parasites ranging from *Ichthyophtirius* to *Apiosoma* and *Trichodina*. *In vivo* work documented a preventive effect, when used in-feed, against *Ichthyophthirius* as well (Jaafar and Buchmann, 2011). Trophonts in the skin were not affected by treatment. The extended half-life in the environment makes the usage questionable from an environmental point of view. Other anticoccidials such as amprolium and salinomycin may show effect against the myxozoan *E. leei* in maricultured bream (Golomazou *et al*., 2006), although the exact mode of action is still to be determined.

### Organophosphates

The aquaculture industry initiated the usage of various types of organophosphates including metrifonate, dichlorvos and azamethiphos at early time points. The mode of action is the inhibition of the acetylcholinesterase in the parasites, whereby the worms get paralysed. Low concentrations (<1 mg L^−1^) have been shown to limit infections with monogeneans such as *Dactylogyrus* in cyprinids (Kabata, 1985), *Pseudodactylogyrus* in eels (Chan and Wu, 1984), crustacean parasites (e.g. *Lernaea* and *Argulus*) in cyprinids and *Lepeophtheirus* in salmon farming. Early warnings against development of anthelmintic resistance in monogeneans were placed by Goven *et al*. (1980) and the extensive usage of the compounds (such as azamethiphos and others) in salmonid mariculture led to fast and well-documented selection of resistant strains of salmon lice (Kaur *et al*., 2016).

### Pyrethroids

Natural extracts of the plant *Chrysanthemum* containing pyrethroids have a strong effect on crustacean parasites such as *Argulus* and were used in classical Chinese fish farming as a parasiticide (Kabata, 1985). Pyrethroids, such as the compound deltamethrin, were also applied against salmon lice in salmonid mariculture but continuous administration induces selection of resistant strains (Bakke *et al*., 2018). The toxicity of the compound to fish calls for precaution when applying these substances.

### Avermectins

The salmon industry has suffered from significant infections by salmon lice *Lepeophtheirus salmonis* since the early start in the late 1970s and early 1980s. The usage of hydrogen peroxide and organophosphates such as azamethiphos and metrifonate (brand names Neguvon and Nuvan) was preferred in the first decades of Norwegian mariculture. The emamectin benzoate (an avermectin product) was introduced in the late 1990s and shown highly effective as convenient in-feed treatment (Stone *et al*., 2002). Other crustacean parasites such as *Argulus* could be controlled in a corresponding way (Hakalahti *et al*., 2004). This accelerated its use until widespread drug resistance appeared in salmon lice (Lees *et al*., 2008), whereafter the industry turned to other ways of control (cleaner fish, mechanical removal, flushing with high-temperature water).

### Benzimidazoles

Mebendazole belongs to the benzimidazole group, which has been used in human and veterinary medicine for decades. A solution of the compound was shown by Szekely and Molnar (1987) to exert a strong and effective effect on the gill monogenean *Pseudodactylogyrus* parasitizing the European eel *Anguilla anguilla*. Following toxicological studies in the laboratory, it was also found effective as a bath in large-scale settings in recirculated eel farms (Buchmann and Bjerregaard, 1990). It was then regularly and extensively used in the aquaculture industry for years despite laboratory experiments warned about the risk for development of anthelmintic resistance (Buchmann *et al*., 1992). Consequently after 6 years, it could be demonstrated that a high degree of anthelmintic resistance occurred at farm level (Waller and Buchmann, 2001). Other benzimidazoles such as flubendazole and albendazole showed effects as well but due to their common mode of action (binding to tubulin monomers) cross resistance is expected to occur due to several common structures of the molecules. Problematic monogenean (*Heterobothrium okamatoi*) infections of maricultured tiger puffer in Japanese waters were previously treated by hydrogen peroxide bathing (Ogawa and Yokoyama, 1998), but a new compound, the pro-benzimidazole febantel, has been the drug of choice for the last decades (Hirazawa *et al*., 2000; Kimura *et al*., 2009). Elimination of external parasites, such as monogeneans, by use of anthelmintics is less complicated because the affected parasites are released from the host surface. However, endoparasites represent another problem. Benzimidazoles have seen a wide application in treatment of livestock nematode infections and fish nematodes such as *Anguillicoloides* (swimbladder nematode) in eels are susceptible as well. However, when treating fish with large nematode burdens in internal organs (such as the swim bladder) special attention should be placed on the risk of excessive antigen liberation from dying worms. The exposure of the host to high nematode antigen concentrations (in organs or systemically) may lead to an exacerbated immuno-pathological reaction.

### Praziquantel

Already four decades ago the anthelmintic praziquantel was found highly effective against the trematode *Schistosoma* and subsequently it was introduced in various aquaculture settings targeting monogeneans (Schmahl and Mehlhorn, 1985; Sitjà-Bobadilla *et al*., 2006), trematodes such as *Diplostomum* eyeflukes (Bylund and Sumari, 1981) and cestodes such as *Bothriocephalus* (Pool *et al*., 1984). The compound is still being applied although lower sensitivities of various parasite species have been reported.

### Antibiotics (fumagillin)

Infections of fish with myxozoans are a major problem in both marine and freshwater. The infections are not readily treated but an antibiotic, termed fumagillin as it is isolated from *Aspergillus fumigatus*, has been shown to prevent development and cyst formation in the fish. Documentation was provided for *Tetracapsuloides bryosalmonae* (PKD agent) in salmonids (Hedrick *et al*., 1988), *Myxidium giardi* in eel (Szekely *et al*., 1988), *Myxobolus* spp. in common carp (Buchmann *et al*., 1993) and *E. leei* in maricultured sharpsnout bream (Golomazou *et al*., 2006).

### Chitin synthesis inhibitors

Arthropods, such as insects and crustaceans (including parasites such as parasitic copepods, isopods and branchiurans), perform several moults during their life cycle, which leaves them vulnerable to compounds inhibiting their formation of a new exoskeleton made of chitin. The chitin synthesis inhibitors comprise several compounds, which have been successfully used against salmon lice infections in Atlantic salmon in Norwegian mariculture and against the isopod *Ceratothoa oestroides* infecting seabass, seabream and meagre in Mediterranean mariculture (Bouboulis *et al*., 2004; Colak *et al*., 2018). Diflubenzuron, hexaflumuron, lufenuron and teflubenzuron all inhibited the transition of the salmon louse from the nauplius to the copepodid stage. The inhibition was associated with a decreased expression of the chitin synthase 1 gene in hexaflumuron and diflubenzuron-treated larvae (Harðardottir *et al*., 2019). Environmental concerns with regard to free-living invertebrates and vertebrates may limit the use of the drugs.

## Mechanical control

### Mechanical filters

The most direct way to limit development of a parasite infection in a host population is to block the life cycle. For certain parasites with free-living infective stages this can be achieved by continuous mechanical filtration of fish tank water. Various mesh sizes of filter screens may be selected to fit the size of the parasite. Thus, tomonts of *Ichthyophthirius* released from the skin of fish generally have a diameter of several hundred micrometres and will be trapped by filters, even with a mesh size of 80 *μm* (Heinecke and Buchmann, 2009). Whenever a tomont is trapped by the filter and removed from the system, then the cyst formation is prevented, and thereby also production of up to 1000 infective theronts within the next 36 h, dependent on temperature. Infective cercariae of eye flukes may correspondingly be trapped by use of mechanical filters (Larsen *et al*., 2005). Filtration of tank water in eel farms using 40 *μ*m filters is able to remove eggs and oncomiracidia of the gill parasitic monogenean *Pseudodactylogyrus anguillae* and *Pseudodactylogyrus bini* (Buchmann, 2012).

### Parasite egg traps

The branchiuran parasite *Argulus* reproduces by laying egg clusters on submerged objects including aquatic plants, branches and roots. Following hatching of *Argulus* eggs, the larvae develop into the infective stage and attach to the fish. This reproductive strategy can be utilized for control. Regular immersion into an infected pond of wooden slats, lattices and bundles of branches to which the female parasite attaches egg clusters provides an opportunity to kill the eggs. This is achieved simply by removing these egg traps from the ponds with few days intervals. New traps are replacing the recovered ones and will be used for parasite oviposition. In this way the reproductive potential of the parasites can be, at least partly, exhausted leading to a decreased infection pressure in the ponds (Kabata, 1985). Submerging boards of various materials into water bodies with problematic *Argulus foliaceus* infections on rainbow trout have been used to reduce the infection level of *Argulus* in rainbow trout lakes (Gault *et al*., 2002). Egg clusters could be harvested regularly when recovering the boards and pulling them ashore. Thereby the overall infection pressure fell and the prevalence and mean intensity decreased 6- to 9-fold.

### Delousing management

Due to the decreasing sensitivity of salmon lice to the different biocides, chemotherapeutants and medicines, which occurred after extensive usage in mariculture farms, alternative control methods had to be developed. These included mechanical removal by heat treatment, brushing or flushing with freshwater. The technique necessitates capture and handling of large salmon, which challenges the health and welfare of the fish (Østevik *et al*., 2022). Other approaches in action are based on laser technology targeting salmon lice on the fish surface. Automated camera systems placed in the water are able to scan passing fish in the netpen and if a salmon louse is detected the laser entity emits a pulse of high energy light towards the louse aiming at killing the parasite *in situ*. Although potentially lethal to the louse, recent controlled full-scale tests could not document a decrease in the mean number of parasites in laser-exposed netpens (Bui *et al*., 2020).

### Farm and netpen construction

New design of netpens applies the use of barriers preventing entrance of infective parasite stages into the section with fish. The upper water layers are preferred by the infective copepodids of the salmon louse. In salmon, mariculture skirts or tarpaulin may be placed around the upper part of the netcages in order to reduce contact between fish and parasites and thereby infections. Thus, the so-called snorkel netcage farms have been developed in order to minimize the attachment of sealice copepodids on maricultured Atlantic salmon. The fish are kept in submerged netpen compartments in the deeper water layers, zones which have reduced abundance of infective louse stages due to the surface-seeking behaviour of copepodids. A lower mean intensity of infection was observed in these cages (Geitung *et al*., 2019).

### Intermediate host control

Eyefluke infections in fish are caused by infective cercariae, released from intermediate host snails, penetrating the surfaces of fish skin, fins or gills (Duan *et al*., 2021). If these cercariae cannot be eliminated by chemicals or mechanical filtration of water (Larsen *et al*., 2005) it is possible to remove the intermediate host snails simply by collecting snails from ponds. As each snail may produce 58 000 cercariae per day this procedure may effectively limit the infection pressure in a pond (Lyholt and Buchmann, 1996).

## Biological control

### Cleaner fish

Natural marine and freshwater ecosystems exhibit a wealth of symbiotic relationships between fish infected with ectoparasites. In tropical fish farming, mosquito fish *Gambusia* feed on the branchiuran parasite *Argulus* during their free-swimming activity (Kabata, 1985) and smaller fish can easily recognize ectoparasites on other often larger fish and pick them of the host skin (Bjordal, 1991; Cowell *et al*., 1993). This basic biological function is being applied by the industry by stocking salmon netpens with cleanerfish. Various species of wrasse have been applied during the latest three decades for removal of salmon lice from salmon skin (Bjordal, 1991; Groner *et al*., 2013; Imsland *et al*., 2014). Their effect is low during wintertime whereas another cleanerfish, lumpsucker *Cyclopterus lumpus*, has a superior performance at low temperatures. A huge industry has been established in order to produce lumpsucker, a species with appetite for salmon louse attached to salmon skin (Groner *et al*., 2013; Imsland *et al*., 2014). Even this sustainable approach is challenged by the high adaptability of salmon lice. Forms with a lower degree of pigmentation, and thereby a lower chance of being recognized and eaten by cleaner fish, have appeared. Both environmental and genetic factors may influence this change of pigmentation (Hamre *et al*., 2021), but these less visible lice are likely to decrease the efficacy of cleaner fish. Various fish species predating on snails (Ben-Ami and Heller, 2001) may be considered a supplement for control of digenean parasites using snails as intermediate hosts. By eliminating snails by predation these fish may contribute to a lowered infection level.

### Cleaner invertebrates

In addition to cleaner fish removing ectoparasites from the surface of infected production fish, a series of other solutions, based on predation by invertebrates, for parasite control exist. The filtration of huge water masses by blue mussels *Mytilus edulis* can be used for trapping the pelagic larval stages (copepodids) of salmon lice *L. salmonis* (Bartsch *et al*., 2013). Free-living copepods such as *Cyclops* predate on fish parasitizing *Diplostomum* cercariae (Bulaev, 1982) and oncomiracidia of *Pseudodactylogyrus* monogeneans (Buchmann, 1988). Likewise free-living turbellarians (*Stephanostomum* sp.) ingest freshly delivered eggs of *Pseudodactylogyrus* whereby the infection level for fish decreases (Buchmann, 2012).

## Immunological control

### Immunostimulants

Stimulation of the immune system of the teleost host by adding various immunestimulants to the feed is a strategy applied by aquaculturists to a wide extent. Slight decreases of infection levels may be recorded following this type of feeding both with regard to protozoans such as *Ichthyophthirius* (Xueqin *et al*., 2012) and metazoans, such as *L. salmonis* (Poley *et al*., 2013). Effects of caprylic acid in combination with iron and mannan (a potential immunostimulant) in feed for seabream in Mediterranean mariculture against monogenean infections (*Sparicotyle chrysophrii*) were recorded by Rigos *et al*. (2016), but the exact mode of action needs to be investigated. However, the efficacy of in-feed immunostimulants for protection of fish against pathogens is generally very low when compared to the effect of vaccination.

### Vaccination

Today vaccination against both bacterial and viral diseases has proven to be the most sustainable ways to control fish disease in aquaculture enterprises. In Europe alone about 1.3 billion fish are successfully vaccinated annually against various infective diseases (Midtlyng, 2022). The marked immune responses established in fish, when infected by various protozoan and metazoan parasites, may lead to some level of protective immunity in fish surviving a natural infection. This was documented for the parasitic ciliates *Ichthyophthirius* (Buschkiel, 1910; Bauer, 1953; Hines and Spira, 1974; Sigh and Buchmann, 2001; Alishahi and Buchmann, 2006) and *Philasterides* (Lamas *et al*., 2008), for the flagellates *Trypanosom*a (Woo, 1981), *Cryptobia* (Jones and Woo, 1987) and *Ichthyobodo* (Chettri *et al*., 2014), for monogeneans such as *Gyrodactylus* (Lindenstrøm and Buchmann, 2000), *Pseudodactylogyrus* (Slotved and Buchmann, 1993) and the crustacean parasite *Lernaea* (Woo and Shariff, 1990). This suggests the existence of a potential for development of antiparasitic fish vaccines (Jørgensen *et al*., 2012), but up until now no such products are licensed for use in commercial aquaculture. Immunological protection of fish against various parasites is likely to be based on a plethora of cellular and humoral (innate and adaptive) elements, which are not so easily induced by a vaccine. So, although no antiparasitic vaccines are available for fish, it may be worthwhile to apply controlled (low to moderate) parasite infections in order to induce a protective response, which may supplement other control strategies. This response induced by a natural infection is likely to include the relevant immune reactions needed to control, at least partly, the host against reinfection.

### Parasitic disease resilience and gut microbiota

The interface between mucosal immunity, gut microbiota and resistance towards parasites has been explored with some success. Stimulation of the gut microbiota by functional feed additives, such as sodium butyrate, may be a way to at least partly control infections with the myxozoan *E. leei* in maricultured seabream (Piazzon *et al*., 2017). It may also be speculated if feed additives such as caprylic acid and mannan exert their effects on the gill monogenean *S. chrysophryi* through a general systemic immune stimulation in seabream (Rigos *et al*., 2016).

## Genetic control through breeding

The ability to resist a parasitic infection is genetically determined, which has been indicated for *Ichthyophthirius* and *Myxobolus* infections in rainbow trout (Hedrick *et al*., 2003; Avila *et al*., 2022). Breeding towards more disease-resistant fish is a possibility, and marker-assisted selective breeding is a tool which has been increasingly used in breeding programmes. Selective breeding of fish carrying certain desirable traits has been in use for decades also in fish aquaculture. The classical approach often takes several years before results are seen, because the generation time of fish can be several years. Several studies have discovered quantitative trait loci (QTL) for viral, bacterial and parasitic diseases. This was demonstrated for infectious pancreatic necrosis virus (Houston *et al*., 2008; Moen *et al*., 2015) and salmonid alpha virus (Aslam *et al*., 2020) in Atlantic salmon and for viral haemorrhagic septicaemia virus resistance in rainbow trout (Verrier *et al*., 2013). Studies have also described QTL associated with resistance in salmonids to bacterial infections caused by *Piscirickettsia salmonis* (Correa *et al*., 2015), *Flavobacterium psychrophilum* (Wiens *et al*., 2013; Vallejo *et al*., 2014) and *Vibrio anguillarum* (Du *et al*., 2011; Wang *et al*., 2014; Shao *et al*., 2015; Karami *et al*., 2020). In addition, the approach is useful for parasite–host systems as well. QTL for resistance towards AGD were described in Atlantic salmon (Robledo *et al*., 2018) and for the scuticociliate *Philasterides dicentrachi* infecting turbot (Rodriguez-Ramilo *et al*., 2013). QTL for resistance in rainbow trout against *Myxobolus cerebralis* was studied by Hedrick *et al*. (2003) and later by Baerwald *et al*. (2011). Investigation of single-nucleotide polymorphism (SNP) markers indicated that some genes associated with resistance towards *I. multifiliis* are located on rainbow trout chromosomes 16 and 17 (Jaafar *et al*., 2020). Innate response genes in the Atlantic salmon were targeted by Gilbey *et al*. (2006) focusing on gyrodactylid monogeneans (*Gyrodactylus salaris*). Likewise, Gharbi *et al*. (2009) and Robledo *et al*. (2019) searched for genes associated with resistance towards salmon lice in this host. Ozaki *et al*. (2013) found corresponding host–parasite associations for the capsalid monogenean *Benedenia seriolae* infecting yellowtail *Seriola quinqueradiata.* Novel typing technology applying markers makes it easier to conduct genotyping. A microarray comprising 57 501 markers (SNP) was used to locate genes encoding resistance to *V. anguillarum* on chromosome 21 (Omy 21) (Karami *et al*., 2020), and genes associated with *Ichthyophthirius* resistance [chromosomes 16 and 17 (Omy 16 and 17)] (Jaafar *et al*., 2020). Genotyped breeders (females and males) carrying the SNPs associated with the favourable trait can easily be selected and used for production of a new generation of trout with a higher innate resistance to infection (Buchmann *et al*., 2022). The basis for this innate and heritable protection may be partly associated with immune factors in the host. However, it cannot be excluded that other elements, including chemoattraction of the parasite to the host, may be involved in better performance of QTL fish.

## Integrated control

Parasites are in general highly adaptable to environmental changes and even a documented control method may in a few years show inferior. An example from the search on compounds with lethal effects on salmon lice is the successful introduction, documentation and victory of the avermectin emamectin benzoate against *L. salmonis* infections. However, its use decreased due to rapid selection of drug-resistant parasite strains. Similar processes apply for organophosphates and pyrethroids. Parasites possess a remarkable ability to adjust to environmental changes as strains with resistance to new conditions are readily selected. In order to secure the fish production from massive infestations of parasites it is worthwhile to introduce integrated control strategies involving several antiparasitic approaches. At present, control of *Ichthyophthirius* infections in freshwater trout farms involves mechanical filtration with micro-sieves (removing tomonts from the water phase), regular addition to fish pond water of biocides/auxiliary substances (hydrogen peroxide, peracetic acid or sodium percarbonate eliminating infective theronts), tolerance of a low initial parasite infection (which induces a relative and partly protective immune response) and use of breeds with an elevated level of natural resistance to infection (Jaafar *et al*., 2020; Buchmann *et al*., 2022).

## Discussion

**Fig. 1.**
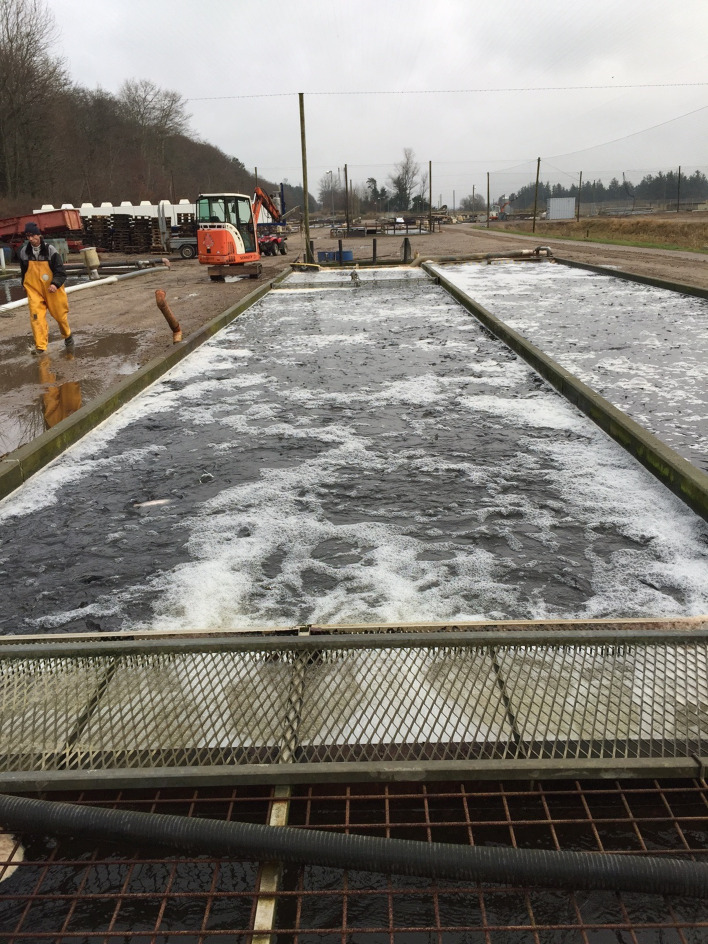
Raceway production of freshwater rainbow trout.

**Fig. 2.**
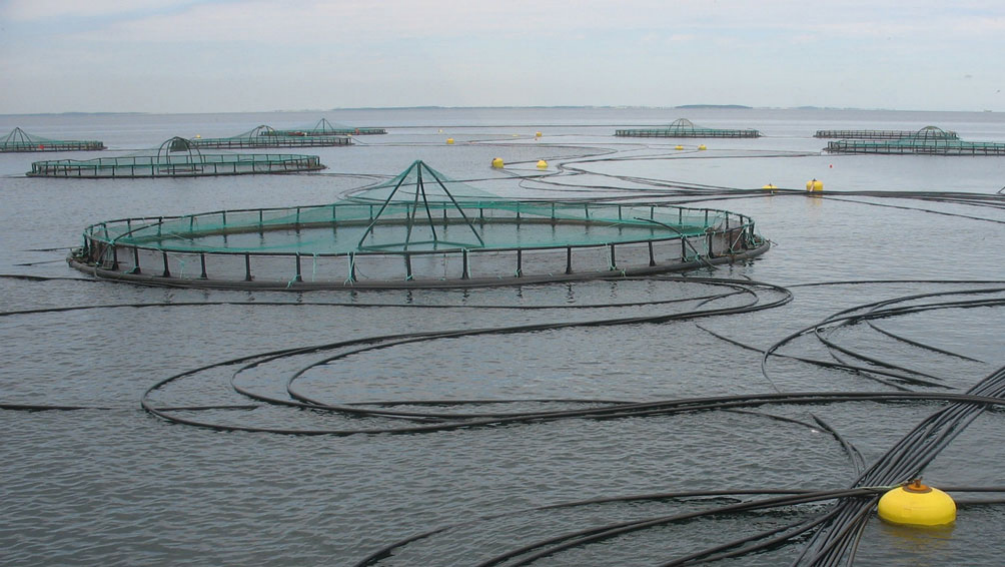
Mariculture farm based on netpen production of rainbow trout in the sea.

**Fig. 3.**
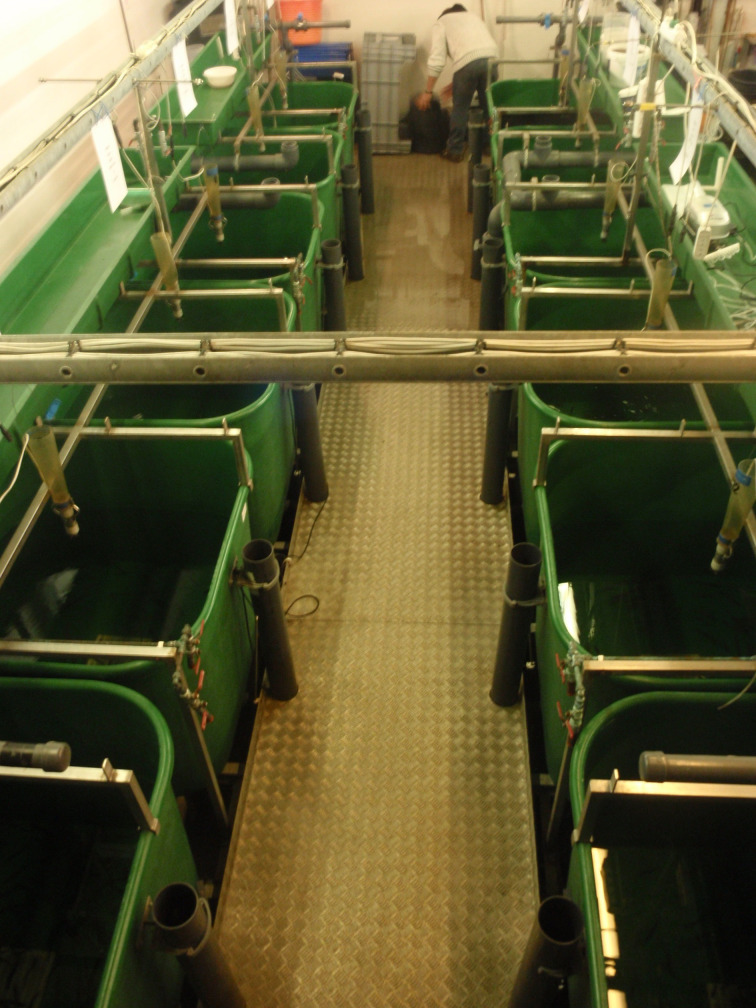
In-door recirculated rainbow trout production.

**Fig. 4.**
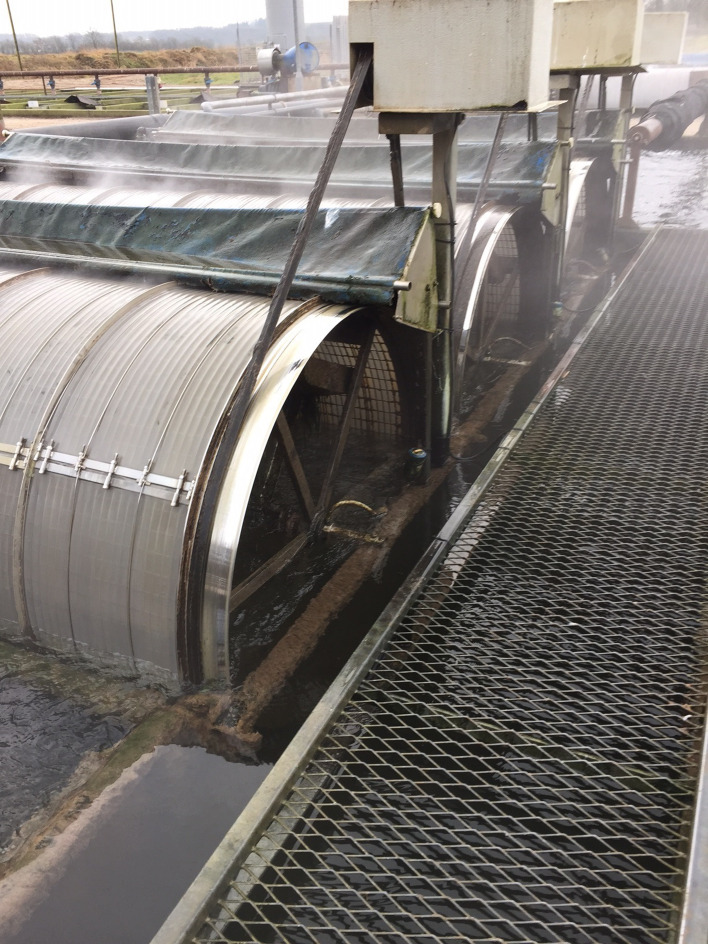
Diagrammatic overview of control elements to be combined into an integrated approach for prevention or treating fish parasite infections in aquacultured fish.

**Fig. 5.**
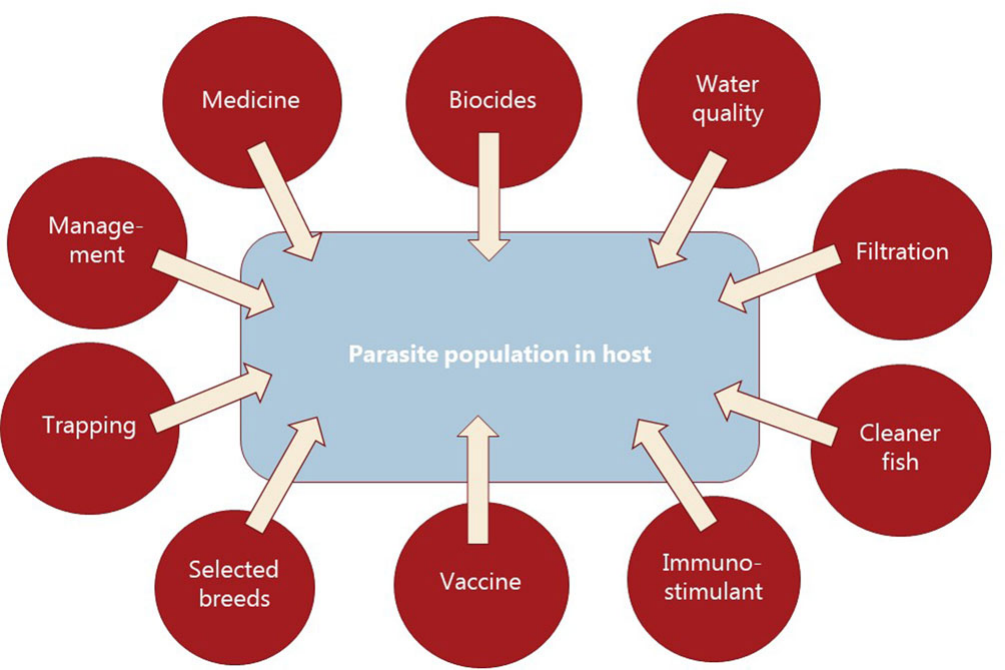
Mechanical filter removing particles in a freshwater trout farm.

Parasitism is one of the most successful life forms on earth. The use of a host for transport, forage area and breeding zone is smart and has therefore been selected by the majority of existing species through evolution. Most free-living host species carry more or less specific parasites and parasites carry in most cases even hyperparasites. Although a parasite seeks to remain undetected on or in the host in order to avoid killing from various host immune element infections they may eventually be confronted with marked host responses. The parasite population may reach levels which elicit injuries and induce pathological and humoral/cellular reactions which expel the parasite from the host. During this process immunosuppressive molecules are often produced by the parasite in order to minimize the host response but this may weaken the host even more and make it susceptible to infections by bacterial and viral pathogens (Kamiya *et al*., 2018). This applies for fish parasites as well. Aquaculture of teleosts is generally based on keeping fish in confined environments ranging from earth or concrete ponds or raceways (Fig. 1), *via* netpens in lakes and marine areas (Fig. 2) to in-house recirculated farms on land (Fig. 3). In all systems parasite infections may be so severe that profitability of the enterprise become endangered due to losses of fish (mortality) or decreased prices from fish products of lower quality (Shinn *et al*., 2015). Aquaculturists must necessarily instigate control measurements, and the present report has briefly introduced various options including chemical, medical, mechanical, genetic and biological control methods. In mariculture it is possible to prevent infection by anisakid nematodes by sequestering fish in e.g. netpens and feed them only heat-treated feed, whereby live nematode larvae cannot reach the fish (Fioravanti *et al*., 2021). In addition, prevention of any introduction of pathogens, including parasites, may be achieved in closed high technology systems with recirculation of water. Disinfection of the entire production system after each production cycle, and combined with introduction only of certified pathogen free and disinfected eggs, will allow running of a disease-free system (Xueqin *et al*., 2012). The use of sterilizing measures such as UV-irradiation (Gratzek *et al*., 1983) and ozonization is able to support such a system. However, the costs associated with such a management practice are high and may challenge profitability. Most systems are therefore facing parasite-induced problems. Experience shows that only one control method is likely to fail at a certain time. This encourages the implementation of an integrated control strategy seeking to combat parasites through multiple targets (Fig. 4). The classical and convenient way to address a newly diagnosed disease is to apply chemotherapeutants and medicines but as mentioned above, the impressive adaptability of parasites may leave these approaches less effective, or near useless, within a few years of intensive usage (Helgesen *et al*., 2015; Kaur *et al*., 2016). Resistance to treatments is easily developed, and increase of the therapeutic dosage is not always possible due to the toxicity of parasiticides to the host organisms (Tedesco *et al*., 2020). In-feed use of immunostimulants for the fish may elevate the resistance to infection marginally but cannot alone sustain health in any production (Akhter *et al*., 2015; Mathiessen *et al*., 2021*a*). Vaccination of fish against bacterial and viral diseases is to a wide extent applicable as documented by a catalogue of vaccine products licensed and currently being used (Midtlyng, 2022). However, effective vaccines against fish parasites are not readily produced (Jørgensen *et al*., 2012), despite available documentation of occurrence of protective immune responses in fish against both protozoan (Woo, 1981; Sigh and Buchmann, 2001) and metazoan parasites (Woo and Shariff, 1990; Lindenstrøm and Buchmann, 2000) following natural infections. More or less controlled infections of farmed fish at a sufficiently low level may induce a level of immunity, which can contribute to an acceptable presence of parasites in farms. The limited number of control methods based on chemicals, drugs and vaccines has forced the industry to develop alternative mechanical techniques, some of which have been used in classical but more primitive fish farming. In this context the use of cleaner fish, which clearly reduces the load of ectoparasites on farmed fish (Bjordal, 1991; Cowell *et al*., 1993), is still an option despite the selection of less pigmented and hardly visible salmon lice is known to challenge their performance (Hamre *et al*., 2021). The exhaustion of the reproductive potential of a population of *Argulus* by regularly harvesting their egg clusters on submerged material is laborious but possible (Kabata, 1985; Gault *et al*., 2002). Correspondingly, removal of parasite stages (ciliates, monogenean eggs, digenean larvae, crustacean larvae) by mechanical filtration (Larsen *et al*., 2005; Heinecke and Buchmann, 2009) or biological mussel filtration (Bartsch *et al*., 2013) has also a potential, although absolute control is not achieved. Another promising strategy is to apply selective breeding of fish with a certain level of natural resistance to infection (Jaafar *et al*., 2020; Avila *et al*., 2022). The adaptability of parasites to any environmental disturbance or challenge makes it necessary to combine the different control systems in order to reduce the selective pressure for occurrence of avoidance mechanisms in parasites. Such an integrated approach has been installed in many types of fish culture systems. In freshwater trout farms mechanical filters (mesh size 40 or 80 *μ*) have been installed for removal of particles (Fig. 5). This will also eliminate a sub-population of the *I. multifiliis* tomonts floating in the water currents and thereby prevent their transformation into the sessile and attached tomocyst stage and their subsequent release of infective theronts (Heinecke and Buchmann, 2009). Removal of tomocysts may also be achieved through suction devices (vacuum cleaning) of fish tanks specifically designed to resist the attachment of tomocysts (Shinn *et al*., 2009). As a supplement the use of regular additions of peracetic acid (Meinelt *et al*., 2009; Bruzio and Buchmann, 2010), sodium percarbonate or hydrogen peroxide reduces the concentration of infective theronts (Heinecke and Buchmann, 2009). Formalin is still being used for the same purpose but its adverse effect on the surface epithelia of the fish may question the use as seen from fish welfare point of view (Buchmann *et al*., 2004; Mathiessen *et al*., 2021*b*). Further, due to the carcinogenicity of formalin it is listed as a human health hazard. The recent documentation of antiparasitic effects of a natural bacterial surfactant released from the bacterium *Pseudomonas* H6 (Li *et al*., 2022) may add this novel compound to the list of possible control agents. As this method is not absolutely effective at the farm level, a sustained parasite infection pressure will occur. However, this may be sufficient to establish a relatively low infection of fish, which will induce a partly protective immune response. Recently, QTL for *I. multifiliis* resistance were described (Jaafar *et al*., 2020), validated (Buchmann *et al*., 2022) and then used for selection of parent fish with a documented better resistance. The specific markers (SNPs) are now being implemented in breeding programmes whereby the resulting new generations carry genes for natural resistance against this particular parasite. The mentioned ways to control parasite occurrence in fish farms have all at a certain time proved effective to some extent. However, the impressive adaptability of both protozoan and metazoan parasites to environmental manipulations in both freshwater and marine aquaculture facilities will in a few years result in parasite strains resisting various treatments if used repeatedly over time. It is therefore recommended to apply an integrated control strategy (Fig. 4). Alternation between control methods will limit the selective pressure for development of resistance to a particular control method. Some techniques may be applied at the same time (e.g. filtration of water to remove *Ichthyophthrius* tomonts and concomitant peracetic water treatment to eliminate theronts). Others may be used in alternation, which on a theoretical basis may be considered to delay selection of parasite strains with resistance to a multitude of control methods.

## Conclusions and future directions

The impressive ability of parasites to adapt to environmental changes challenges an effective and lasting control of parasitoses in aquaculture settings. Even novel compounds with high efficacy may be ineffective within a few years of constant usage at farm level. It is recommended that aquaculturists should combine various methods in an integrated control strategy. Alternation between different chemical, medical, biological and mechanical control methods may delay development of resistance. Continued research into basic biology and biochemistry of the parasites can lead to novel approaches replacing old and less effective methods. Up until now no effective control method based on hyperparasitism has been documented in aquaculture settings. Hyperparasites occur in natural ecosystems and future research should show if this could be a supplementary tool in fish parasite control.

## Data Availability

Data are available on request.
